# Monthly Summary

**Published:** 1882-09

**Authors:** 


### MONTHLY SUMMARY.

---

*American Dyspepsia.*—In the Boston *Medical and Surgical Journal*, Dr. James H. Robbins takes the ground that the prevalence of dyspepsia among us is due less to improper food, badly prepared, to taste in eating, or to excessive eating, than to the fact that we nearly all are the possessors, either by inheritance or acquirement, of imperfect nervous organizations that are consequently unable to lend their aid to digestion as they should; for this reason but few of us are able to digest enough food for our bodily repair; hence we experience the sensation of having over-eaten, when in reality we have not had enough. The author says:—

"Such causes operate with special power upon us of the present generation of the Anglo Saxon race, whose ancestor came to this country several generation ago, for the reason that from these ancestors we inherit nervous systems of impaired vigor. Our parents and grandparents were so strenuously engaged in an arduous, ambitious, and competitive endeavor, amid the rapidly changing condition of life in a new country, to secure competency and respectable social position, that they expended to a great degree, their own vital powers, and have accordingly transmitted to their children delicate and neurotic constitutions. If it is granted that we inherit a diminished vitality, it is easy to see how ingeniously the pressure, the wear and tear, the strain, the excitement, the hurry and worry incident to this breathless, bustling life of ours, affect the nervous system. It is also evident that impairment of digestion and mal-nutrition must be the certain result of such a condition of things."

This deterioration of the nervous system is fostered and increased by our faulty methods of raising children and our own erroneous style of living when we reach maturity. The evident remedy is to be found in an elevation of the tone of the nervous system. Whether this theory be true or not, it is a very charming one, and would certainly seem to account for much of the dyspepsia that renders so many persons miserable.—*Medical and Surgical Reporter.*

---

*Rest in Treatment of Heart Disease.*—By this we mean not positive, but comparative rest; neither do we refer to acute inflammatory affections of the heart, wherein, from the very gravity of the disease, confinement to bed and consequent rest become necessarily assured. We are thinking of those cases of heart exhaustion, so to speak, of individual whose general health and tonicity is much run down, from overwork or abuse, and in whom the heart shares in this general vitiation. Possibly the organ is not in itself diseased; its organic integrity may be perfect, but its muscular walls may be flabby and weak, ready to yield to, or, more properly, unable to resist any great strain. If when in this condition, the man resorts to any violent muscular exercise, or subject himself to the influence of violent physical emotions, this weak heart may become mechanically distended in its efforts to perform the extra labor demanded of it. Or it may be that dilatation has already taken place to some extent; then does it become important to allow the organ time for the development of the beneficent hypertrophy that will do so much to preserve its integrity. By rest we mean to advise your patients who are threatened with or already have dilatation of the heart to do everything slowly, to perform every act of life deliberately, and to avoid, as far as possible, all occasions calculated to excite the passions emotions. We must ever remember what a delicate machine the heart is and how easily it can become deranged, and realizing this, must consider how much more care this organ requires when it is already diseased. We must, under such circumstances, walk slowly think slowly, eat slowly, in a word, do everything slowly. It is not well, and we do not recommend the carrying of this advice to the verge of laziness; but what we do mean is, that while it is well for all (either sound or diseased) to avoid hurry, it is ten times more important, aye, absolutely imperative, for the man with a weak or diseased heart.—*Medical and Surgical Reporter.*

---

*Food Adulteration.*—Dr. Charles Smart, U. S. A., in the *National Board of Health Bulletin*, January 1st, 1881, submits a report of interest on this subject, prepared by instructions from the National Board of Health. The first adulterated article of food alluded to is tea. Of 109 teas examined, 90 were obtained from sources which should have furnished a pure article, 19 were obtained from questionable sources. The results of thorugh examinations permits the committee to report that they think tea adulteration is not carried on to any very great extent. In

England, the chief adulteration of coffee is chicory. Dr. Hassall testified before the Food Commission, in 1855, that of 34 samples which he had examined, 31 contained chicory. Normandy found as much as 75 per cent. of chicory. Roasted wheat, bean rhy and potato flours, mangel-wurzel and a substance resembling acorns, roasted corn, and ground cocoa-ribs, while as adulterations of the chicory were found deal and mahogany sawdust carrots, Venetian red and burnt sugar. The reporter pertinently remarks that while in this country we have the credit of making wooden nutmegs, we have never been accused of manufacturing coffee beans from compressed chicory. In England, sand in sugar is a familiar phrase. Most of the sugar there examined was dark in color, mixed with much vegetable extractive, and swarming with sugar-mite. The sugar of the present day is purer. The report concludes that manufacturers of flour do not to any great extent doctor the flour, but that bakers do. Corn meal, bicarbonate of soda, cream of tartar, baking powders, black pepper, pickles, confectionery, and other common household agents, were noticed, and a detail of the method of examining them. It is a useful report.—*Buffalo Medical and Surgical Journal.*

---

*Tobacco Smoking—An Experiment*—A writer in the *British Medical Journal* says: "If the fumes from a cigar, pipe or cigarette be instantly ejected from the mouth and throat before descending into the chest, and be made to pass through a cambric handkerchief drawn tightly across the open lips, a permanent deep yellow stain, corresponding in size and shape to the opening between the lips, and having numerous spots of a darker hue pervading it, will be left on the handkerchief; but that the prolonged puff from the chest, after inhalation from a cigarette, fails under similar circumstances to produce any but a scarcely perceptible and speedily evanescent mark. Query: What in the latter case becomes of the substance which stains?

"I am not aware of any instances on record in which the lungs of the cigarette-smokers have been specially examined; but it would be interesting to know whether by perseverance one could color one's bronchial tubes as one does a meerschaum; and, if so, whether the process would be attended with risk."—*Pacific Med. and Surg. Journal.*

*A Marvel of Surgery.*—Dr. Roswell Park, of chicago reports a marvelous case that he has recently seen in Prague. The great surgeon, Gussenbauer, more than a year ago removed the larynx and epiglottis of a patient suffering from cancer. Six weeks afterward he began to wear part of the artificial larynx, and after accustoming himself to this, he gradually learned how to introduce and use the reed which takes the place of the vocal cords. The patient is a riding teacher, talking a great deal, and does not suffer the slightest inconvenience or pain. His voice is monotonous, but his enunciation excellent, his speech perfectly intelligible, and he eats and drinks with peafect facility. Three intra-laryngeal operations had been previously made before Gussenbauer attempted his feat.—*Medical and Surgical Reporter.*

---

*Nitrous Oxide.*—Further experience has not changed the relative position or very much enlarged the sphere of action of nitrous oxide. That is the safest of all anæsthetics has been established beyond a question. In one institution where such administration is subject of record, this gas has been given over 100,000 times, and not only without a death but without causing in a single instance symptoms sufficiently serious to necessitate transporting the patient home in a carriage. In the city of Philadelphia alone, it has been given over 133,000 times without a death, and without any injuries results. Death cannot be justly attributed to it in more than four cases since its intrcduction.—*F. C. Reeve, in Holmes' Surgery, American Edition.*

---

*Oxalate of Lime Calculus.*—Dr. E. L. Keyers presented the calculus material removed by rapid lithotrity from the bladder of a boy, nineteen years of age, who was referred to him by Dr. Vondy, of Jersey City. It weighed four hundred and forty-three grains, and he thought that the entire stone weighed one ounce It was pure *oxalate of lime.*. The operation occupied thirty-two minutes. The largest grasp was one inch and a fourth. Although he used a two-inch wheel—unusually large—the stone was crushed with a good deal of difficulty. The debris was removed without trouble through a No. 16 tube. It was the largest oxalate of lime calculus he had ever encountered. —*Med. Record.*

---

*Backache: Its Causes.*—Dr George Johnson, in (*Brit. Med. Journal,*) says that in the great majority of cases the pain of backache has its seat in the muscles, and is a simple result of strain or over fatigue of the lumbar and erectores spinal muscles and tendons. A remarkable feature of the pain resulting from excessive muscular exercise is that, while it may continue more or less during rest in bed, it is usually much increased by the first movements after rest, but gradually diminishes after moderate exercise. In muscular lumbago, standing is more fatiguing for the back and legs than walking, and leaning forward puts a greater strain on the muscles of the back than standing erect. Pain is often more severe on one side than on the other, owing to the common practice of throwing the weight more on one leg than on the other. A common cause of painful overstrain in the dorsal muscles is an excessive weight in the abdomen, whether from advanced pregnancy, dropsy, or excessive development of fat. Dr. Johnson, in speaking of these causes, incidentally gives the dietary advisable in obesity. He also calls attention to dyspeptic myalgia resulting from malnutrition of the muscle. "Growing pains," Dr. Johnson thinks, are due to excessive muscular exercise, and are to be cured by rest. Sudden pain is sometimes caused by cramp or rupture of some fibers of the muscle during contraction. Indigestible food sometimes causes cramps in muscles in certain individuals instead of cramps in the stomach. Cold, as is well known, is a freqnent cause of pains in other muscles besides those of the back. For lumbago Dr. Johnson recommends hot air or Turkish baths, followed by vigorous shampooing; also an embrocation composed of equal parts of linimentum belladonnæ and linimentum opii.

Other causes of backache are aneurism of the aorta, the symptoms of which Dr. Johnson gives at some length, with illustrative cases; cancerous glands in the abdomen; diseases of the kidneys; gastric ulcer; uterine diseases; diseases of the bones of the spine and of the spinal cord; and, finally, the backache of commencing fevers.

